# Qualitative and Quantitative Multiplexed Proteomic Analysis of Complex Yeast Protein Fractions That Modulate the Assembly of the Yeast Prion Sup35p

**DOI:** 10.1371/journal.pone.0023659

**Published:** 2011-09-13

**Authors:** Virginie Redeker, Chris Hughes, Jimmy Savistchenko, Johannes P. C. Vissers, Ronald Melki

**Affiliations:** 1 Laboratoire d'Enzymologie et Biochimie Structurales, Centre National de la Recherche Scientifique, Gif-sur-Yvette, France; 2 Waters Corporation, Atlas Park, Manchester, United Kingdom; University of Akron, United States of America

## Abstract

**Background:**

The aggregation of the baker's yeast prion Sup35p is at the origin of the transmissible [*PSI^+^*] trait. We and others have shown that molecular chaperones modulate Sup35p aggregation. However, other protein classes might be involved in [*PSI^+^*] formation.

**Results:**

We designed a functional proteomic study that combines two techniques to identify modulators of Sup35p aggregation and describe the changes associated to [*PSI^+^*] formation. The first allows measuring the effect of fractionated *Saccharomyces cerevisiae* cytosolic extracts from [*PSI^+^*] and [*psi^−^*] yeast cells on Sup35p assembly. The second is a multiplex qualitative and quantitative comparison of protein composition of active and inactive fractions using a gel-free and label-free LC-MS approach. We identify changes in proteins involved in translation, folding, degradation, oxido-reduction and metabolic processes.

**Conclusion:**

Our functional proteomic study provides the first inventory list of over 300 proteins that directly or indirectly affect Sup35p aggregation and [*PSI^+^*] formation. Our results highlight the complexity of the cellular changes accompanying [*PSI^+^*] formation and pave the way for *in vitro* studies aimed to document the effect of individual and/or combinations of proteins identified here, susceptible of affecting Sup35p assembly.

## Introduction

Infectious proteins (prions) aggregation is at the origin of neurodegeneration in higher vertebrates [1]. Prions have indeed the ability to undergo conformational conversion from a functional constitutive form to non-functional and toxic, high molecular weight species that recruit functional prions and convert them to the non-functional forms [2]. Thus, prion aggregation is a self-perpetuating process and the breakage of the aggregates into smaller aggregates contributes to an amplification of the aggregation process [3].

Prions are at the origin of dominant phenotypic traits that are inherited in a non-Mendelian manner and are transmissible by cytoduction in the yeast *Saccharomyces cerevisiae* [4]. Three such traits are actively studied: [*PSI^+^*], [*URE3*] and [*PIN^+^*]. They are due to the aggregation of the proteins Sup35, Ure2 and Rnq1, respectively. These proteins readily assemble under physiological salt conditions and neutral pH into fibrillar high molecular weight particles [5]–[8]. Yeast prions N-terminal moieties that are essential for prion aggregation and propagation are unusually rich in glutamine and asparagine residues. They resemble in that to huntingtin which aggregation is involved in the neurodegenerative Huntington's disease. Thus, yeast prions constitute good models to document the mechanism of protein aggregation and conformational conversion propagation observed in conformational neurological diseases.

The eukaryotic release factor Sup35p, also known as eRF3, mediates together with Sup45p (eRF1), ribosomal translation termination [9]. In [*PSI^+^*] cells, the aggregation of Sup35p alters translation termination and leads to an increased tendency of the ribosomes to read through nonsense ochre stop codons [10].

*In vivo*, the propagation of [*PSI^+^*] is highly dependent on the expression of molecular chaperones. We recently documented *in vitro* the assembly of Sup35p alone and in the presence of molecular chaperones from the Hsp40, Hsp70 and Hsp100 families alone or in concert, and showed that molecular chaperones finely tune the aggregation of Sup35p [11]. Indeed, while the yeast Hsp70 Ssa1p, together with its Hsp40 co-chaperones Sis1p or Ydj1p was shown to sequester Sup35p, in an ATP-dependent manner, in assembly incompetent oligomeric species, Hsp104p was shown to stimulate Sup35p nucleation and polymerization [11]. We also documented the functional interplay between chaperones and demonstrated that Ssa1p together with Sis1p or Ydj1p and ATP counteract the assembly stimulatory effect of Hsp104p.

Classical proteomic, approaches including aggregates purification and immune precipitation, 2D gel electrophoresis and mass spectrometric identification of proteins, have been recently used to document the changes in protein expression profiles accompanying cell degeneration in a number of neurodegenerative diseases [12]–[16], including prion disease [17]. More recently, mass spectrometric based strategies, combining the identification and quantification of proteins have been used to perform a global quantitative proteomic analysis of a Drosophila model of Parkinson disease [18]. These approaches led to the identification of specific proteins and altered functional protein families and protein networks.

Efficient analysis of large amounts of raw data for peptide and protein identification and quantification in complex protein mixtures is a challenge in mass spectrometry-based proteomic approaches. Two strategies have been developed to overcome difficulties. In one approach, labels are incorporated within the peptides and proteins; in the other no label is used [19]. The use of labels, based on the principle of stable isotope dilution theory, introduces mass tags that can be incorporated metabolically, chemically or enzymatically. Chemical label strategies include isotope coded affinity tags (ICAT) [20], or isobaric tags such as iTRAQ [21], which involve the use of a derivatization reagent for chemical modification of proteins in a site-specific manner. These labels are chemically identical within the peptides from two (or more) samples and will thus present identical chromatographic properties and ionization efficiency, allowing different samples to be analyzed and quantified simultaneously by mass spectrometry. In label-free methods, quantification is obtained by directly correlating the MS signal intensity and the relative or absolute protein quantity. This can be achieved either by a spectral counting approach, using MS/MS acquired data and counting the number of fragment spectra leading to protein identification [22], [23], or by comparative analysis of precursor ion intensities [24], [25]. Among the label-free approaches, the data-independent LC-MS^E^ method provides accurate mass information on both the precursor and their associated fragment ions, in low and elevated energy mode, respectively, whilst concurrently recording the intensity of both ion types. This label-free LC-MS method allows the identification of proteolytic peptides over a relatively high dynamic range and protein quantification via normalization of the LC-MS datasets through comparison of the peptide intensities across multiple data sets [25], [26]. An addition to the scanning method includes the molar amount determination for each identified protein, using the intensity peptide ratio from a given protein to that of a reference [27].

To identify modulators of the prion Sup35p conversion, we have developed a functional proteomic study. First, we have fractionated *Saccharomyces cerevisiae* extracts from [*PSI^+^*] and [*psi^−^*] cells using a sucrose gradient. The effect of each fraction was tested on Sup35p assembly *in vitro*. Two fractions exhibiting significant differences on Sup35p polymerization were selected as they might contain protein factors involved in [*PSI^+^*] propagation. In order to determine qualitative and quantitative changes in the protein composition of the different fractions, we performed a multiplex comparison between the fractions using a proteomic analysis based on a gel-free and label-free LC-MS approach. We obtained interesting protein profiles for each fraction and were able to observe not only qualitative but also quantitative differences between proteins of each of these fractions. This first functional proteomic study using a data-independent LC-MS scanning method reveals interesting changes in proteins belonging to the protein folding and/or protein degradation pathways and emphasizes the role of some functional protein families in prion propagation.

## Materials and Methods

### Expression and purification of Sup35p

Sup35p was overexpressed in *E. coli* strain BL21-CodonPlus, in 2×YT media complemented with chloramphenicol (34 µg/ml) and carbenicillin (100 µg/ml), at 30°C. At OD_600_ = 0.5–0.7, protein expression was induced with 1 mM IPTG. The bacterial pellets were resuspended in 20 mM Tris–HCl, pH 8.0, 1 M NaCl, 20 mM imidazole, 5 mM β-mercaptoethanol, 5% glycerol, supplemented with EDTA-free protease inhibitor cocktail tablets (Complete, Roche Diagnostics Gmbh, Mannheim, Germany). After disruption of the cells by sonication, Sup35p was purified and stored in 50 mM Tris-HCl, pH 8.0, 1 M NaCl, 5 mM ß-mercaptoethanol, 5% glycerol, 10 mM MgCl2 and 2 mM EGTA at −80°C as described [11]. Sup35p was dialyzed for 1h30 against assembly buffer (50 mM Tris-HCl, pH 8.0, 200 mM NaCl, 5% glycerol, 5 mM ß-mercaptoethanol, 5 mM GTP, 10 mM MgCl_2_).

### Cytosolic Yeast extract fractionation

74D-694 *[MAT alpha, ade1-14(UGA), trp1-289(UGA), his3* Δ *- 200, ura3-52, leu2-3, 112]* [*PSI^+^, PIN^+^*], and 74D-694 *[MAT alpha, ade1-14(UGA), trp1-289(UGA), his3* Δ *- 200, ura3-52, leu2-3, 112]* [*psi^−^, PIN^+^*] yeast strains were grown in YPD medium supplemented with adenine 0.75 mM to an optical density at 600 nm of 2–4. The cells were spun (2,000 RPM at 4°C for 5 minutes, JLA8.1000 rotor, Beckman Instruments, Inc., California) and rapidly washed twice with water. A volume of glass beads (0.45–0.5 mm, B. Braun biotech international, GmbH, Germany) equal to that of the final pellets (2 ml) was added to the pellets and 2 ml of the protein extraction buffer (25 mM Tris HCl, pH 7.5, 50 mM KCl, 10 mM MgCl_2_, 1 mM EDTA supplemented with Complete protease inhibitors, Roche Diagnostics, GmbH, Germany, 1 caplet for 25 ml) were added. The cells were then harvested by vigorous shaking (8 shaking cycles of 30 seconds each spaced by 30 second pauses on ice). The mixture was then spun (3,000 RPM at 4°C for 5 min) and the supernatant (2 ml) recovered and loaded within 20 min on premade 60-20% sucrose gradients. The tubes were immediately inserted in a Beckman SW41TI rotor and spun at 77,000 g for 4.5 h at 4°C using an Optima L90K Beckman ultracentrifuge (Beckman Instruments, Inc., Brea, CA). The rotor was allowed to stop without a break. 1 ml fractions were collected from the bottom to the top of each tube and labeled fraction 1 (bottom of the tube, 60% sucrose) to 8 (top of the tube, 0% sucrose). The fractions were aliquoted, flash frozen and stored at −80°C.

The sucrose gradients were prepared by loading 1.4 ml layers of sucrose 60, 50, 40, 30 and 20% in 14×89 mm polyallomer centrifuge tubes (Beckman Instruments, Inc.). The tubes were then frozen and thawed prior to use.

### Assembly of Sup35p into protein fibrils

Sup35p assembly reactions were monitored as described [11] using thioflavin T binding [28]. When assembly was performed in the presence of fractionated cytosolic yeast extract, the different fractions were dialyzed against assembly buffer prior to the addition of soluble Sup35p (7 µM). The final fractionated cell extract concentration in the assembly reaction was 0.48 mg/ml.

### Protein digestion

The protein fractions were dialyzed against 50 mM ammonium bicarbonate (pH 8.5) for 2 h and spun 15 min at 14,500 RPM and 4°C. The pellets were discarded and the protein concentrations in the supernatants determined using the Bradford assay [29]. High purity bovine serum albumin (BSA, Sigma-Adrich, St. Louis, MO) was added as an internal standard. This internal standard allows monitoring experimental variation during sample digestion and LC-MS measurements. The protein concentrations were adjusted to a final concentration of 200 fmoles/µl of BSA and 0.4 mg/ml of the sucrose gradient fractions in presence of 0.1% RapiGest (Waters corporation, Milford, MA) [30]. The proteins were then reduced in the presence of 5 mM dithiothreitol at 56°C for 30 min and alkylated in 15 mM iodoacetamide in the dark at room temperature for 30 min. Protein digestion was performed at 37°C, overnight, using trypsin (Promega, Madison, WI) at a protein to protease ratio of 50∶1 (w∶w). Trypsin digestion and RapiGest treatment were stopped by the addition of an equal volume of 500 mM HCl followed by incubation at 37°C for 45 min. The tryptic peptide samples (0.2 µg/µl of total yeast protein and 50 fmoles/µl of BSA) were spun for 10 min at 14,500 RPM. The supernatant was rapidly frozen and stored at −80°C until further analysis.

### Liquid chromatography - mass spectrometry

Reversed-phase separation of the tryptic peptides was performed using a nanoAcquity system (Waters Corporation, Milford, MA) equipped with a 180 µm×20 mm Symmetry C18 5 µm trap column and a 75 µm×200 mm BEH C18 1.7 µm analytical column. Solvent A was water with 0.1% formic acid. Solvent B was acetonitrile with 0.1% formic acid. Peptides were eluted from the column using the following gradient at a flow rate of 300 nl/min: 3 to 40% solvent B in 90 min, 40 to 90% solvent B in 1 min, 90% B for 4 min and re-equilibration with 3% solvent B for 20 min (Figure S2). The lock mass solution, comprising [Glu^1^]-Fibrinopeptide B in 0.1% formic acid in water/acetonitrile (75∶25, v/v), was delivered by the auxiliary pump of the nanoAcquity system to the reference sprayer of the NanoLockSpray source at a concentration of 200 fmoles/µl. The samples were mixed with an equal volume of a solution containing 25 fmoles/µl of Phosphorylase B predigested with trypsin (Waters Corporation, Manchester, UK). Pre-digested Phosphorylase B was added as a technical LC-MS variation internal standard. Finally, 2.5 µl of the digested samples were loaded on the column with 3% of solvent B, corresponding to 250 ng of total protein digest, 62.5 fmoles BSA and 31.25 fmoles Phosphorylase B. Triplicate measurements were performed for each sample. Mass spectrometric analysis of the tryptic peptides was performed with a Q-TOF Premier mass spectrometer (Waters Corporation, Manchester, UK). The instrument was operated in data independent, alternate scanning (LC-MS^E^) acquisition mode. The acquisition time for each mode was 1 s. At low energy, the collision energy was kept constant at 4 eV, whilst at elevated energy the collision energy was ramped from 15 to 35 eV during the acquisition. The interscan delay time was 0.1 s. The cone voltage was set at 24 V to prevent in-source fragmentation and the *m/z* acquisition range was from 50 to 1990 for both acquisition modes.

Biological replicates, corresponding to independent cell cultures, extracts preparation and fractionation, denoted fractions 4B [*psi^−^*], [*PSI^+^*] and 6B [*PSI^+^*], were analyzed as described above.

### Data processing: Protein identification and quantification

The nanoscale LC-MS data were processed and searched using ProteinLynx GlobalSERVER (PLGS) v2.3 (Waters Corporation). The qualitative part of the software utilizes the physicochemical properties of polypeptides and statistical models [31]. Protein identification data are presented in Table S1 (and Table S5A for independent biological replicates). Quantitative label-free LC-MS analyses are described and justified in detail in the results section ‘Qualitative and quantitative multiplexed proteomic analysis of cytosolic sucrose fractions from [*PSI^+^*] and [*psi^−^*] by LC-MS’. The detailed identification and quantification procedure is presented in File S1.

### Isoform processing

The data were processed manually for protein paralogs. For homologous proteins, the observed signal intensity arising from sequences common to different polypeptides can result in redundant identifications. This is advantageous from a qualitative perspective since the intensity of the redundant peptides is cumulative. However, this leads to difficulties in quantitatively assessing individual protein paralogs. When isoform specific peptide sequences were detected, a quantification of protein isoforms was carried out. If not, the different isoforms were reported as a single protein homology group and an absolute amount assigned to the group as a whole. In the case of the chaperones Ssa1-4 and Sse1-2, a novel filtering method (Table S6), using an extension to the earlier presented absolute quantification scheme, was applied. Namely, the average intensity was calculated for every isoform proteotypic peptide. The proteotypic peptide intensities were subsequently used to segment the total intensity of the common peptide belonging to each parent protein. Next, the peptides were re-ordered based on their segmented intensities for the common sequences and non-segmented intensities for the proteotypic peptides and the molar amounts calculated. When a given peptide was not identified by the software, its intensity information was retrieved manually using its accurate mass and retention time.

Biological functions of the identified proteins were completed following Gene ontology annotation at http://db.yeastgenome.org/cgi-bin/GO/goSlimMapper.pl.

## Results

### *[PSI^+^]* and *[psi^−^]* cytosolic fractions promote the assembly of Sup35 into fibrils

**Figure 1:**
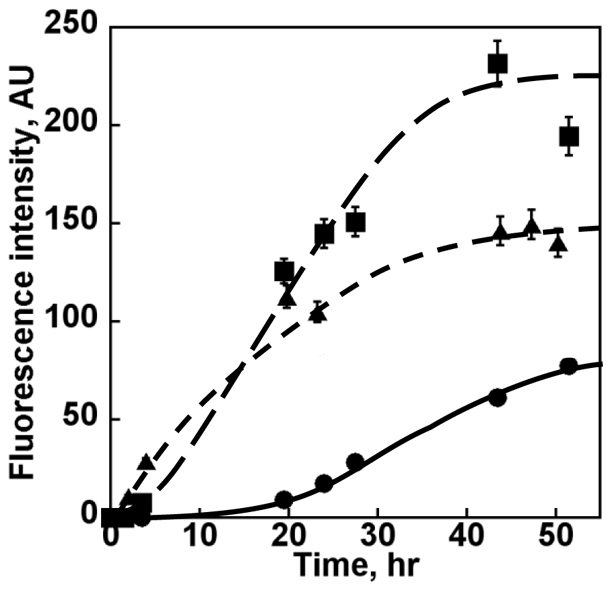
Assembly of full-length Sup35p into protein fibrils in the presence of cytosolic fractions from [*PSI^+^*] and [*psi^−^*] cells. Soluble Sup35p (7 µM) was incubated at 10°C in assembly buffer with no addition (solid circles) or containing 0.48 mg/ml of fraction 4 from [*PSI^+^*] (solid squares), or [*psi^−^*] (solid triangles) cells. The time course of Sup35p assembly in the presence of cytosolic fraction 6 from [*PSI^+^*] or [*psi^−^*] cells superimposes to that with no addition. Statistic analysis of the differences in fluorescence intensity between fractions was made using a Student T-test calculation. The P values were 0.03, 0.017 and 0.6 for fraction 4 [*PSI^+^*] versus fraction 6 [*PSI^+^*], fraction 4 [*psi^−^*] versus fraction 6 [*PSI^+^*] and fraction 4 [*psi^−^*] versus fraction 4 [*PSI^+^*] comparisons, respectively.

To identify regulators of Sup35p assembly, total cytosolic extracts from [*PSI^+^*] and [*psi^−^*] yeast strains were prepared and fractionated on 20–60% sucrose gradients. The effects of these fractions after dialysis on Sup35p were monitored using thioflavin T binding (Figure 1 and Figure S1 for independent biological replicates).

Most fractions exhibited limited or no effect on Sup35p assembly, as for example fraction 6 from [*PSI^+^*] or [*psi^−^*] (20–30% sucrose, containing protein complexes of 1.62 to 7.6 MDa). Fractions 4 however, (40% sucrose, containing protein complexes of 35.6 to 167 MDa) from both yeast strains affected the lag phase preceding the assembly of Sup35p into fibrils, diminishing it from ∼15 hours in the control reaction to 1–4 hours. Moreover, thioflavin T fluorescence intensity at steady state increased very significantly, although to different extents, in the presence of proteins present in fractions 4 from both yeast extracts suggesting an increase in the amount of assembled Sup35p. Interestingly, the extent of thioflavin T fluorescence increase was higher in the presence of fraction 4 from [*PSI^+^*] than from [*psi^−^*] cells while the nucleation phase was shorter in the presence of fraction 4 from [*psi^−^*] than from [*PSI^+^*] cells. This effect is not due to the extracts as no increase of thioflavin T fluorescence is observed for extracts incubated under the same conditions without exogenous Sup35p.

We conclude from these observations that the cytosol of [*PSI^+^*] and [*psi^−^*] yeast strains differ by the factors that modulate the assembly of Sup35p into protein fibrils. To unveil these differences, the protein content of fractions 4 from [*PSI^+^*] and [*psi^−^*] yeast strains were compared. As fractions 6 from both yeast strains are devoid of an assembly stimulatory activity, these protein fractions can be used as a control. We therefore also compared the protein content of fractions 4 and 6.

### Qualitative and quantitative multiplex proteomic analysis of cytosolic fractions from *[PSI^+^]* and *[psi^−^]* yeasts by LC-MS

**Figure 2:**
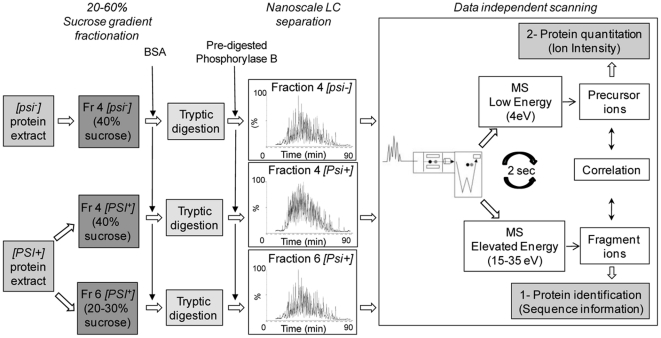
Strategy for qualitative and quantitative proteomic analysis of proteins involved in regulation of Sup35p assembly. This scheme summarizes the different steps of the experimental strategy detailed in materials and methods Section, from yeast protein extracts to the qualitative and quantitative LC-MS analysis.

A comprehensive multiplex comparison of the protein composition of fractions 4 from [*PSI^+^*] and [*psi^−^*] cell extracts and of fractions 4 and 6 from [*PSI^+^*] cell extract was performed in order to identify modulators of Sup35p assembly. This proteomic comparison was done after adjusting the protein concentrations to the same value following the LC-MS approach described in Figure 2 and the Materials and Methods section. The samples were spiked with BSA prior to trypsin digestion and with Phosphorylase B prior to LC-MS measurement. These two internal standards were used to normalize the data and calculate variations between samples. Tryptic peptides eluted from the LC system were analyzed on-line in a multiplexed fashion as described in the Materials and Methods section. The low energy acquisition trace in Figure S2 shows a continuous measurement of the m/z values of all the eluted and ionized tryptic peptide ions at a given time point of the separation process. The high energy acquisition trace collected in parallel comprises the fragmentation products of those peptides. An inventory of peptide precursors along with their time-resolved fragment ions was generated. The use of a novel database search strategy designed for data independent acquisitions, together with the accurate mass measurements of both precursors and fragment ions, the high reproducibility of the chromatographic separation, the time alignment of the m/z values of both the precursors and the associated fragmentation ions obtained at high energy, allows identification of proteins with high confidence [31], [32]. The analysis of the deconvoluted low energy m/z data of all the precursor peptides over an entire chromatographic peak allows quantification of peptides and proteins in large scale proteomic studies.

Such analyses rely on the hypothesis that most of the proteins within the samples that are compared are similar. This is the case when proteins present within a given fraction from [*psi^−^*] and [*PSI^+^*] cell extracts (e.g. fractions 4) are compared. However, significant differences in the protein composition of fractions are expected when different fractions from one sucrose gradient (e.g. fraction 4 and fraction 6 from [*PSI^+^*]) are compared. In the latter case, the LC-MS approach requires a normalization procedure between samples using an internal standard. This was addressed, in this study, by expressing the amounts of a protein of interest as a fraction of the total amount of proteins per individual fraction and experiment. This multiplexed approach allows not only the identification of a large number of proteins over a high dynamic range, but also the quantitative comparison of several protein sets associated to different biological activities, even when the protein composition is vastly different.

**Figure 3:**
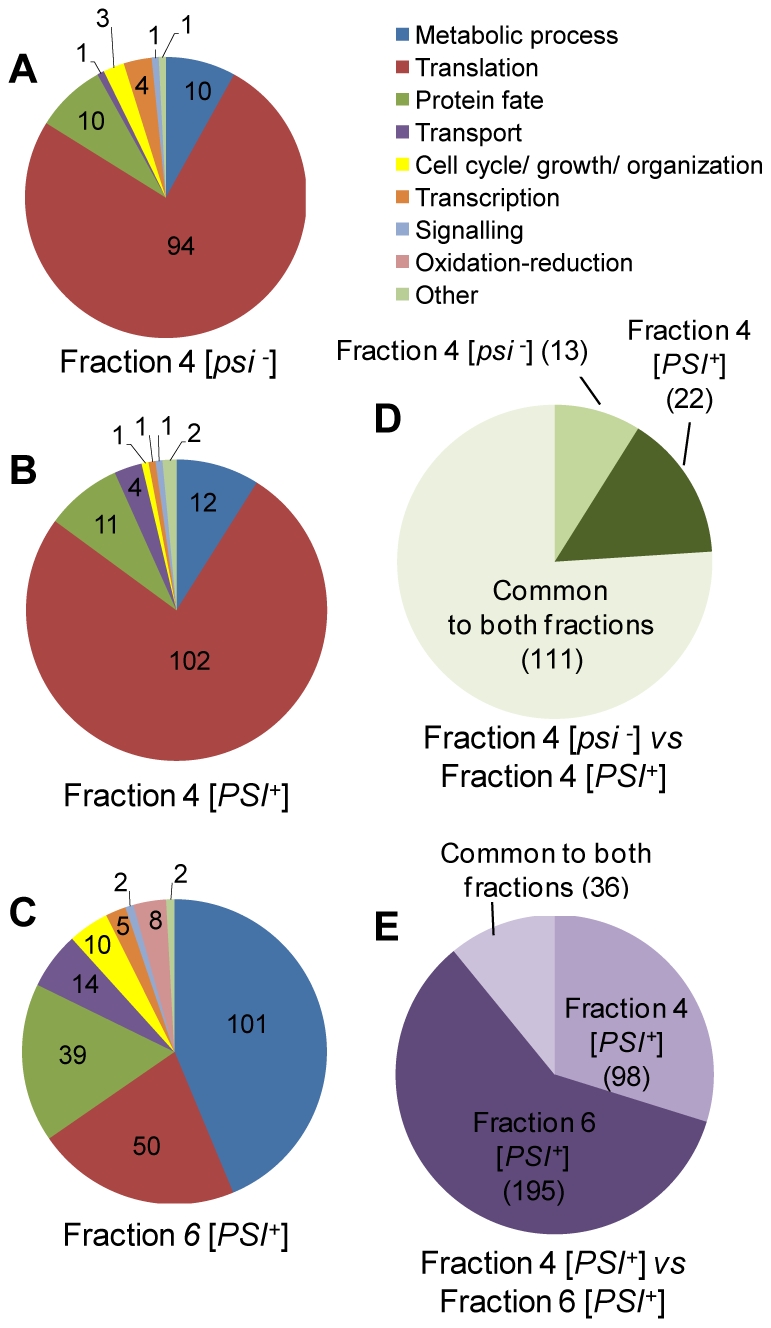
Qualitative comparison of proteins identified in the selected sucrose gradient fractions. (A), (B) and (C) represent the distribution of the major biological process of each of the 124, 134 and 231 proteins identified in fractions 4 from [*psi^−^*] cells, fraction 4 from [*PSI^+^*] cells and fraction 6 from [*PSI^+^*] cells. (D) and (E) illustrate the qualitative comparison between fractions 4 [*psi^−^*] versus fraction 4 [*PSI^+^*] and fraction 4 [*PSI^+^*] versus fraction 6 [*PSI^+^*], respectively.

In the qualitative analysis, the protein composition of the three fractions were compared (fractions 4 from [*psi^−^*] and [*PSI^+^*] and fraction 6 from [*PSI^+^*]) using a single-dimension reversed phase gradient separation (Figure S2) and the detection and identification process previously reported [32]. The list of the identified yeast proteins is presented in Table S2. The number of proteins identified in at least two out of three technical replicates for fraction 4 [*psi^−^*], 4 [*PSI^+^*] and 6 [*PSI^+^*] is 124, 134 and 231, respectively. The reproducibility of protein identification is about 80%. The biological processes where each identified protein is involved were determined using gene ontology annotation and are given. Whereas, the highest number of identified proteins are involved in metabolic and protein translation processes, a third category of proteins of interest is involved in protein folding and modification (Figure 3 A, B and C). The changes for proteins belonging to the latter category within the three fractions we compared are represented (Figure 3 D and E).

In the multiplex quantitative analysis, proteins were quantified using their composite MS signal response as developed by Silva et al [32], [33]. The search algorithm and the normalization procedure are described in the Materials and Methods section and File S1. We determined the individual concentration of the 340 yeast proteins that were identified in at least two replicates (Table S2). The protein concentrations ranged from 0.03 ng/µl to 11.11 ng/µl. As an example, the quantitative analysis of actin and chaperones Ssb1/2 was validated by Western blot analysis (Figure S3). A 40% change in protein concentration was considered as significant.

Our strategy allowed identifying protein fractions composition and quantitative changes in [*PSI^+^*] and [*psi^−^*] fractions composition.

### Comparison of *[PSI^+^]* and *[psi^−^]* protein fractions that promote Sup35p assembly

We first compared the protein composition of fractions 4 from [*PSI^+^*] and [*psi^−^*] cells. Both fractions promote Sup35p assembly but to a different extent (Figure 1). The proteins we identified and quantified in these fractions are listed in Table S2 and compared in Table S3 (and Table S5B and S5C for independent biological replicates) and the biological processes distribution in which they are involved is presented in Figure 3 (and Figure S4 for independent biological replicates. Over 90% of the proteins identified in fractions 4 from [*psi^−^*] and [*PSI^+^*] are involved in protein translation (94 and 102 proteins, respectively), in protein fate, including protein folding, degradation and modification (10 and 11 proteins, respectively) and in metabolic processes (10 and 12 proteins, respectively). A number of proteins such as elongation factor Tef1, ribosomal proteins, Hsp70 chaperones Ssb1/2 and guanine nucleotide binding protein Asc1 were abundant (from 0.5 to 5 ng/µl) in both fractions 4. Additional proteins, such as aspartate transcarbamylase Ura2 and the elongation factors Eft1 and Yef3 are abundant in fraction 4 from [*PSI^+^*].

Over seventy ribosomal proteins constituting the 40S and the 60S ribosome moieties: subunits Rps 0 to 31 and subunits Rpl1 to 43 together with Rpp0 were identified in fractions 4 from [*psi^−^*] and [*PSI^+^*] cells and their measured concentrations were similar (0.6 to 1 ng/µl). As a functional ribosome, composed of one 40S and one 60S, has an approximate mass of 4.2 MDa and given that proteins with molecular masses of 35 to 167 MDa are expected in fraction 4 of our sucrose gradients (40% sucrose), the ribosomes we detected most probably form polysomes composed of several ribosomes interacting with mRNA molecules and associated proteins or protein complexes. Amongst associated complexes, we have identified the Ribosome-associated complex (RAC) involved in the biogenesis of newly synthesized polypeptides. This complex corresponds to a Ribosome-anchored chaperone network composed of the Hsp40 Zuotin (Zuo1) and the Hsp70 Ssz1 interacting with Ssb1/2 [34] and has been reported as a potent antagonist of Sup35p prionogenesis [35]. We also identified yeast Rpl31 that acts as a contact point between RAC and the large ribosomal subunit at the polypeptide tunnel exit [34]. The concentrations of RAC complex subunits (0.47, 0.78 and 2 ng/µl+/−0.14 ng/µl for Zuo1, Ssz1 and Ssb1/2, respectively) suggest that a significant fraction of Ssb1/2 remains available for other protein-protein interactions. We also identified Egd1/Egd2 that form the NAC complex (nascent polypeptide-associated complex) which interacts with nascent polypeptide chains, the RAC complex and proteins from the Hsp70 family. A limited number of proteins involved in protein folding and degradation processes were identified in fractions 4 from [*psi^−^*] and [*PSI^+^*] cells. Ssb chaperone proteins (i.e. Ssb1/2) were particularly abundant in both fractions (about 2 ng, i.e. 2% of the total protein content). Chaperones from the Hsp70 family (Ssa1/Ssa2/Ssa3/Ssa4, Ssb1/Ssb2 and Ssz1) were also detected.

**Figure 4:**
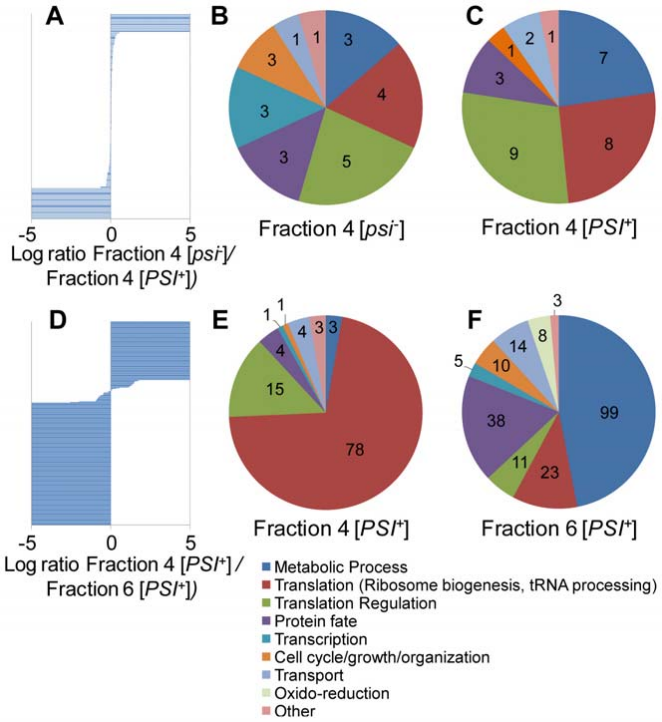
Differential distribution of proteins for which concentrations differs between the selected fractions. (A) and (D) correspond to the comparison of proteins identified in fraction 4 [*psi^−^*] versus fraction 4 [*PSI^+^*] and in fraction 4 [*PSI^+^*] versus fraction 6 [*PSI^+^*] respectively. (B) and (C) represent the distribution of the major biological processes attributed to proteins increased in fraction 4 [*psi^−^*] and fraction 4 [*PSI^+^*] respectively. 23 and 31 unique or significantly increased proteins are present within fractions 4 [*psi^−^*] and [*PSI^+^*], respectively. (E) and (F) represent the distribution of the major biological processes attributed to proteins increased in fraction 4 [*PSI^+^*] and fraction 6 [*PSI^+^*] respectively. 109 and 211 unique or significantly increased proteins are present within fractions 4 and 6 from [*PSI^+^*] cells, respectively.

**Figure 5:**
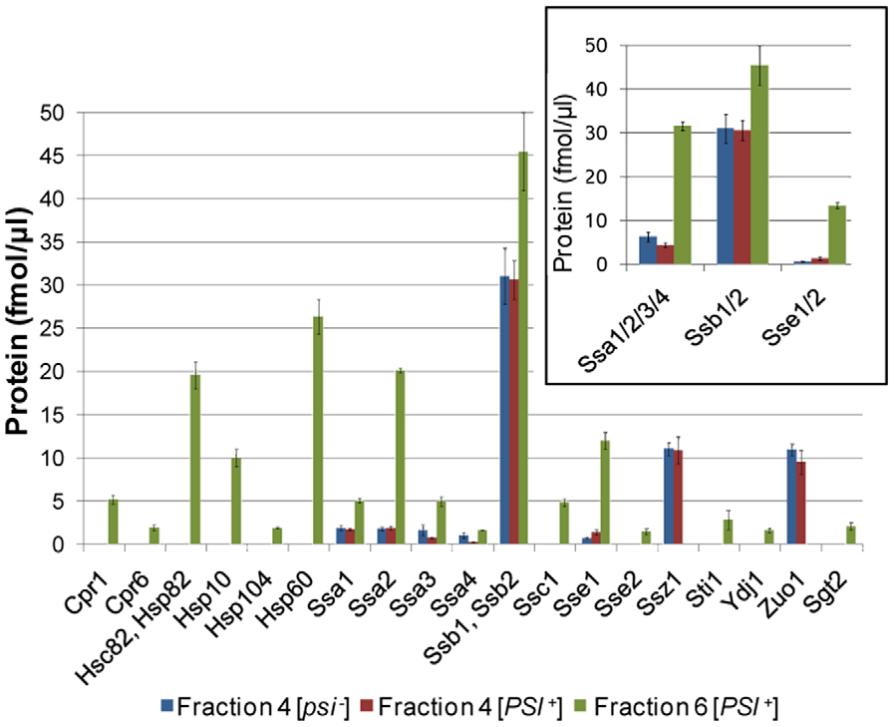
Quantification of cytosolic proteins involved in protein folding. The protein amounts in fraction 4 [*psi^−^*] (blue), fraction 4 [*PSI^+^*] (red) and fraction 6 [*PSI^+^*] (green) were determined as explained in Materals and Methods section. The protein amounts are femtomoles of proteins measured in one microliter of the fractions. The sum of Ssa, Ssb and Sse isoforms is shown in the inset. Error bars correspond to technical variation.

The differential distribution of proteins for which concentration differs in fraction 4 from [*psi^−^*] and [*PSI^+^*] cells is shown in Figure 4. Most of the 23 proteins for which expression levels are increased in fraction 4 from [*psi^−^*] cells are involved in different steps of the protein translation process (Dbp1, Egd2, Nop7, Sro9, Cic1, Gcd11, Tif4631, Pab1 and Sup45, and Rps15), in different metabolic processes (Pda2, Imd1/2/3/4, Lat1), in protein folding (Ssa3, Ssa4) and in other functional processes (Fks1, Kem1, Scp160, Vps1, Yra1, Pma1/2). The 31 proteins for which expression levels are increased in fraction 4 from [*PSI^+^*] cover five classes of biological processes: protein translation initiation (Tif5, Tif32, Nip1, Prt1, Rpg1) and elongation (Hef3, Eif2A, Eft1, Yef3), ribosome biogenesis necessary for protein translation (Rpl42A, Rps29A, Rps30A, Rps31, Rps25A, Rps8A, Mrt4, Arx1), metabolic process (Adh1/2, Ilv5/5G, Tdh1/2/3, Pfk1, Pfk2, Fas1, Fas2), transport processes (New1, Nop3), protein folding (Sse1) and protein modifications (Nat1, Ubi3). Similar observations were made with independent biological replicates (Figure S5). Changes in the concentration of the molecular chaperones we identified within fractions 4 from [*psi^−^*] and [*PSI^+^*] cells appear limited, as shown in Figure 5.

### Comparison of protein fractions from *[PSI^+^]* cells that affect or not Sup35p assembly

To further identify modulators of Sup35p assembly, we compared the protein content of fractions from [*PSI^+^*] cells that affect or not Sup35p assembly, fractions 4 and 6, respectively (Table S4, and S5D for independent biological replicates). The number of proteins identified in fraction 6 from [*PSI^+^*] was much higher than that in fraction 4 in independent biological replicates (Figure 3E and Figure S4E). Over 80% of the proteins identified in fraction 6 are involved in processes ranging from metabolic (101 proteins) to transport (14 proteins), cell growth (10 proteins) and protein translation (50 proteins). Proteins involved in protein folding (18 proteins), degradation (17 proteins) and modifications (4 proteins) processes represent 16% of the proteins identified (Figure 3). Comparison of the protein contents of fractions 6 and 4 from [*PSI^+^*] cells shows that they differ significantly. While proteins involved in metabolic processes (e.g. biosynthesis of amino acids, lipids, purines, cofactors, RNAs and nucleosides, carbohydrate metabolism and glycolysis) represent 9% of the proteins in fraction 4, they represent 44% of the proteins in fraction 6 and about 60% of total protein weight. These proteins are either unique to fraction 6 or their concentration is significantly higher in this fraction. About 20% of the proteins within fraction 6 (25% of total protein weight), are involved in protein translation processes (20 tRNA amino acid synthetases or acylases, 19 proteins involved in regulation of initiation and elongation of the protein translation process as well as 14 ribosomal proteins, 8 of which belonging to the 40S ribosomal subunit). The functional partner of Sup35p, the eukaryotic peptide chain release factor eRF1, or Sup45p, was detected only in fraction 4.

Except Rpl40A (a fusion protein of Ubiquitin and ribosomal protein L40), identified only in fraction 6, ribosomal proteins represent 5% (0.8% of total protein mass) of the proteins in fraction 6 while they represent 55% of the proteins (75% of total protein mass) in fraction 4. In contrast, tRNA amino acid synthetases are exclusively identified in fraction 6. The most interesting differences were observed for proteins involved in protein fate, including protein folding, protein degradation and protein modifications. Only 11 proteins are involved within these biological processes in fraction 4, while 39 proteins lie within this category in fraction 6 (Figure 3 and Table 1). Seventeen out of the 39 proteins we identified are constituents of the Ubiquitin Proteasomal System (6 regulatory subunits from the 19S Proteasome complex with 5 Rpn and 1 Rpt subunits, and 10 subunits from the 20S Proteasome Core with 7 Pre, 2 Pup subunits and Scl1) as well as Ubiquitin. None of these proteins are present in fraction 4 from [*PSI^+^*] or [*psi^−^*] cells. Finally, their concentrations ranged from 0.04 to 0.1 ng/µl.

**Table 1:** Multiplexed quantitative data of proteins involved in protein degradation and modification.

				Fraction 4 [*psi* ^−^]				Fraction 4 [*PSI^+^*]				Fraction 6 [*PSI^+^*]			
Accession n°	Accession name	Protein name	Gene name	n rep	n pep	ng/µl	RSD	n rep	n pep	ng/µl	RSD	n rep	n pep	ng/µl	RSD
P38779	CIC1_YEAST	Proteasome-interacting protein CIC1	CIC1	**2**	**4.50**	**0.04**	**0.35**	0				0			
P21242	PSA3_YEAST	Proteasome component C1	PRE10	0				0				**2**	**6.00**	**0.05**	**0.01**
P30656	PSB5_YEAST	Proteasome component PRE2	PRE2	0				0				**3**	**4.67**	**0.05**	**0.39**
P38624	PSB6_YEAST	Proteasome component PRE3	PRE3	0				0				**3**	**5.00**	**0.07**	**0.62**
P40302	PSA1_YEAST	Proteasome component PRE5	PRE5	0				0				**3**	**9.00**	**0.09**	**0.07**
P40303	PSA7_YEAST	Proteasome component PRE6	PRE6	0				0				**2**	**6.00**	**0.13**	**0.01**
P23724	PSB1_YEAST	Proteasome component C5	PRE7	0				0				**3**	**3.00**	**0.06**	**0.27**
P23638	PSA4_YEAST	Proteasome component Y13	PRE9	0				0				**3**	**4.33**	**0.06**	**0.27**
P32379	PSA5_YEAST	Proteasome component PUP2	PUP2	0				0				**3**	**7.00**	**0.11**	**0.14**
P25451	PSB3_YEAST	Proteasome component PUP3	PUP3	0				0				**3**	**2.00**	**0.04**	**0.01**
P43588	RPN11_YEAST	26S proteasome regulatory subunit RPN11	RPN11	0				0				**2**	**3.50**	**0.06**	**0.64**
Q12250	RPN5_YEAST	26S proteasome regulatory subunit RPN5	RPN5	0				0				**2**	**6.50**	**0.07**	**0.40**
Q12377	RPN6_YEAST	26S proteasome regulatory subunit RPN6	RPN6	0				0				**2**	**6.50**	**0.09**	**0.31**
Q06103	RPN7_YEAST	26S proteasome regulatory subunit RPN7	RPN7	0				0				**2**	**4.00**	**0.03**	**0.01**
Q08723	RPN8_YEAST	26S proteasome regulatory subunit RPN8	RPN8	0				0				**2**	**5.00**	**0.06**	**0.13**
Q01939	PRS8_YEAST	26S protease regulatory subunit 8 homolog	RPT6	0				0				**2**	**7.50**	**0.06**	**0.01**
P21243	PSA6_YEAST	Proteasome component C7-alpha	SCL1	0				0				**3**	**6.67**	**0.06**	**0.09**
P12945	NAT1_YEAST	N-terminal acetyltransferase A complex subunit NAT1	NAT1	0				2	**2.50**	**0.07**	**0.01**	0			
P41940	MPG1_YEAST	Mannose-1-phosphate guanosyltransferase	MPG1	3	7.33	0.15	0.31	3	10.00	0.21	0.27	**3**	**14.00**	**0.65**	**0.09**
P61864	UBIQ_YEAST	Ubiquitin	UBI1	0				0				**3**	**3.67**	**0.10**	**0.10**
P05759	RS37_YEAST	40S ribosomal protein S31	UBI3	1				3	**3.67**	**0.38**	**0.03**	0			
A7A0L9	A7A0L9_YEAS7	Poly-ubiquitin	UBI4	0				0				**3**	**3.67**	**0.50**	**0.11**
P43616	CPGL_YEAST	Glutamate carboxypeptidase-like protein	YFR044C	0				0				**2**	**4.50**	**0.07**	**0.11**

Accession number, accession name, protein name and gene name are indicated for each identified protein. For each analyzed fraction, the number of replicates in which the protein was identified (n rep) is indicated, together with the number of identified peptides (n pep), the average amount of protein identified in one microliter (ng/µl), and the relative standard deviation (RSD).

Among the chaperone proteins, in addition to Ssa1-4, Ssb1/2 and Sse1 identified in fraction 4, the chaperones and co-chaperones Sse2, Ssc1, Ydj1, Hsp60, Hsc82, Hsp104, Hsp10 and Cpr1, Cpr6, Sti1 and Sgt2 which are involved in protein folding, were only detected in fraction 6 (Figure 5). Some of these identified protein chaperones were validated by Western blotting (Figure S3). While Ssb1/2 concentrations were similar, Ssa2 concentration was one order of magnitude higher in fraction 6 than in fractions 4. Zuo1 and Ssz1 were not detected within fraction 6. This suggests that the ribosome-associated RAC complex is absent from fraction 6.

## Discussion

The cytosolic protein fractionation coupled to the functional studies and qualitative and quantitative multiplexed label-free proteomic analysis we present here was intended to identify proteins that modulate Sup35p assembly and [*PSI^+^*] emergence and propagation in yeast cells.

Comparison of the protein content of cytosolic fractions that promote Sup35p assembly to different extents (fractions 4 from [*psi^−^*] and [*PSI^+^*] cells) reveals discrete differences. These fractions are rich in proteins involved in protein translation and folding and in metabolic processes. Proteins found only in fraction 4 from [*PSI^+^*] cells can be related to changes in biological processes within [*PSI^+^*] yeast cells due to the prion phenotype. The major changes we observed affect metabolic pathways and cellular proteostasis that may lead to changes in protein synthesis, folding and degradation.

Comparison of the protein content of cytosolic fractions that have no effect on Sup35p assembly and that promote Sup35p assembly (fractions 6 and 4 from [*PSI^+^*] cells) reveals significant changes. These differences concern proteins involved in protein translation, folding and degradation. They also concern protein involved in oxido-reduction and metabolic processes. We previously documented the functional interplay between Hsp104, 70 and 40 family members [11]. The effects of cytosolic fractions on Sup35p assembly we report here underline the importance of molecular chaperone interplay. Our analysis also provides the first inventory list of chaperones possibly involved in [*PSI^+^*] propagation and/or changes in cellular proteostasis due to the [*PSI^+^*] element.

### Proteins involved in polypeptide folding

Molecular chaperones facilitate the folding of newly synthesized proteins, are involved in protein quality control, assist the assembly of several macromolecular complexes and limit protein aggregation [36]. Several Hsp70 family members (Ssa1, Ssa2, Ssa3, Ssa4, Ssb1/2 and Ssz1) and one co-chaperone Hsp40 (Zuo1) were identified in [*PSI^+^*] and [*psi^−^*] cytosolic fractions (fraction 4) that favor Sup35p assembly. Ssa1 and Ssa2 are constitutively expressed chaperones that bind to denatured proteins and prevent aggregation, while Ssa3 and Ssa4 are heat-inducible. Ssb1 interacts with two co-chaperones to form the ribosome-associated Ssb1∶Zuo1∶Ssz1 complex [37], recently identified as a potent inhibitor of Sup35p assembly and antagonist of prionogenesis [35]. Fractions 4 from [*PSI^+^*] and from [*psi^−^*] cells promote Sup35p assembly. This indicates that protein modulators tune the inhibitory activity of the RAC complex. Indeed, our observations suggest either that the RAC complex does not act post-translationally or is unavailable because of its interaction with polysomes within these fractions. This finding illustrates the importance of performing a global analysis such as that presented here.

The proteins involved in protein folding we identified in fraction 6 from [*PSI^+^*] cells were either unique to this fraction or present at higher concentration than in fraction 4 from the same yeast extract with the exception of Zuo1 and Ssz1, specific to fractions 4, and Ssb1/2 at similar concentrations in both fractions. The nineteen proteins we identified, amongst which one small Heat shock protein from the GroES chaperonin family (Hsp10), one Hsp40 homologue (Ydj1), one Hsp60, Hsp70 homologues (Ssa1, Ssa2, Ssa3, Ssa4, Ssb1/2, Ssc1, Sse1, Sse2), Hsp90 homologues (Hsp82/Hsc82) and one Hsp100 homologue (Hsp104), represent 11 ng (i.e. 8% of the total protein weight).

### Proteins involved in polypeptide degradation

The 26S proteasome is a 2.5 MDa multi-subunit intracellular protease [38] that eliminates proteins, in particular misfolded proteins, targeted for degradation. The 26S proteasome consists of the 20S proteolytic core complex and two 19S regulatory subunits [39].

We have identified ten subunits of the 20S proteasome: six alpha subunits (Pre5, 6, 9, 10, Pup2 and Scl1) and four beta subunits (Pre2, 3, 7 and Pup3), as well as six subunits of the 19S proteasome: five subunits of the lid (Rpn5, 6, 7, 8, 11) and one subunit of the base (Rpt6), in fraction 6 but not in fraction 4 from [*PSI^+^*] cell extracts.

### Proteins involved in polypeptide modifications

Ubiquitin was identified in fractions 4 and 6 from [*PSI^+^*] cells. Ubiquitin is covalently attached to lysine residues either as a monomer or as a polymer. Unlike mono-ubiquitination, poly-ubiquitination is believed to target polypeptides to the proteasome. The overall concentration of ubiquitin in fractions 4 and 6 was similar (0.38 and 0.60 ng/µl, respectively). However, while Ubi3, a poly-protein with one ubiquitin fused to the ribosomal protein S37 was identified in fraction 4, Ubi1 a poly-protein with one ubiquitin fused to the ribosomal protein L40 and Ubi4 a poly-protein containing five repeats of ubiquitin were found in fraction 6.

In addition, the N-terminal acetyltransferase NatA complex (Nat1), critical for the normal function of proteins that need being acetylated, was only found in fraction 4.

### Proteins that affect cellular proteostasis

Proteins involved in protein translation, ribosomal proteins, and/or proteins regulating initiation, elongation or termination of protein translation, differed considerably both in fractions 4 from [*psi^−^*] and [*PSI^+^*] cells and in fractions 4 and 6. Fractions 4 from [*psi^−^*] and [*PSI^+^*] were enriched in polysomes. Interestingly, the ribosome possesses an intrinsic protein folding activity [40]–[42] of which down-regulation destabilizes prion traits [43].

Regulatory factors involved in initiation, elongation and/or termination of protein translation, were overrepresented in fractions 4 from [*PSI^+^*] as compared to that from [*psi^−^*] cells. Sup35p partner, Sup45p (i.e. eRF1) was detected in fractions 4 from [*psi^−^*] and [*PSI^+^*] cells but not in fractions 6. Several translation initiation factors, such as Tif32, Prt1, Nip1, Tif34 and Tif35 that form the core complex of the translation initiation factor 3 complex eIF-3 were identified in fraction 4 from [*PSI^+^*] cells. Finally, the poly(A) binding protein- Pab1 [44] was detected in all fractions.

### Proteins involved in other cellular processes

Proteins involved in metabolic processes represent 60% of total protein mass in fractions 6 from [*PSI^+^*] (about 82 ng of proteins). Tdh1/Tdh2/Tdh3 (Glyceraldehyde-3-phosphate dehydrogenase), Pyk1 (pyruvate kinase) and Pdc (pyruvate decarboxylase) represent each about 5% of the total protein content.

Proteins specifically involved in oxido-reduction processes such as the thioredoxins Trx1 and Trx2 and the thioredoxin reductase Trr1 were found in fraction 6 from [*PSI^+^*] cells but not in fraction 6 from [*psi^−^*] cells.

Our analysis shows that the protein content of cytosolic fractions from [*PSI^+^*] and [*psi^−^*] cells that promote Sup35p assembly into protein fibrils to different extents differs significantly. It is tempting to relate the functional differences we observe to the protein content of the cytosolic fractions. Molecular chaperones modulate [*PSI^+^*] propagation in an exquisite manner [45]–[50] and Sup35p assembly into fibrils [11], [35]. Given that Ssb1/2 are present at similar concentrations in cytosolic fractions 4 and 6, the Sup35p assembly promotion we observe for fraction 4 cannot be attributed to these chaperones. With a similar reasoning, one reaches the conclusion that the effect we observe must be due to a compromized interplay between molecular chaperones that distinguish fractions 4 from 6 either because of their absence or lower concentrations in fraction 4. The following molecular chaperones are good candidates: Hsp10, Ydj1, Hsp60, Ydj1, Hsp60, Ssa1, Ssa2, Ssa3, Ssa4, Ssc1, Sse1, Sse2, Hsp82/Hsc82, Sgt2 and Hsp104, as they either lack or are present at considerably lower concentrations in fractions 4. The importance of a functional interplay between chaperones in Sup35p assembly was previously demonstrated by the analysis of the effect of protein chaperones added individually or in combination to Sup35 assembly assays [11]. Two chaperones (Zuo1 and Ssz1) are specific to fractions 4. These chaperones are probably present within these fractions as a Ssb1∶Zuo1∶Ssz1 complex associated to the ribosomes (RAC). They are therefore unlikely to be responsible for the Sup35p assembly induction we observe.

The finding that fractions 4 are devoid of proteasome in contrast with fractions 6 suggests that the Sup35p assembly promotion we observe is not due to a proteasome-mediated cleavage of Sup35p with generation of protein fragments with increased nucleation ability that could serve as nuclei for exogenous full-length Sup35p assembly. In addition, in fractions 6, the presence of Ubi4, which contributes to protein poly-ubiquitination, together with the proteasome further suggest that Sup35p eventual poly-ubiquitination and proteasome-mediated degradation do not contribute to increased assembly and nucleation ability.

Finally, our analysis unveiled a number of changes within cellular proteostasis that are the consequence of [*PSI^+^*]. Indeed, while proteins involved in polypeptide translation, ribosomal proteins, and/or proteins regulating initiation, elongation or termination of protein translation, differed considerably in fractions 4 from [*psi^−^*] and [*PSI^+^*], proteins involved in oxido-reduction processes were present in fraction 6 from [*PSI^+^*] cells but not in fraction 6 from [*psi^−^*] cells.

Our study provides the first inventory list of over 40 proteins involved in polypeptide folding, degradation and modification that directly or indirectly affect Sup35p assembly into protein fibrils. We also bring evidence for changes within the expression of proteins involved in metabolic processes and the regulation of proteostasis upon wild-type yeast conversion into [*PSI^+^*]. Altogether, our results highlight the complexity of the cellular changes accompanying [*PSI^+^*] formation. This complexity can only be further deciphered by *in vitro* studies where the effect of individual and/or combinations of proteins susceptible of affecting Sup35p assembly will be tested. Further studies destined to assess the effect on Sup35p assembly of the molecular chaperones we identified through this study and their interplay need being conducted. The role of the proteasome and Sup35p degradation in the absence or the presence of a variety of molecular chaperones on [*PSI^+^*] propagation and the assembly propensity of Sup35p and its degradation products need being analyzed.

## Supporting Information

Figure S1 **Assembly of full-length Sup35p into protein fibrils in the presence of cytosolic fractions from** ***[PSI^+^]*** **and [** ***psi^−^*** **] cells from an independent cell extract preparation and fractionation (biological replicates).** Soluble Sup35p (7 µM) was incubated at 10°C in assembly buffer with no addition (solid circles) or containing 0.48 mg/ml of fraction 4B from [*PSI^+^*] (solid squares), or [*psi^−^*] (solid triangles) cells. The time course of Sup35p assembly in the presence of cytosolic fraction 6B from [*PSI^+^*] or [*psi^−^*] cells superimposes to that with no addition.
(TIF)

Figure S2 **Reversed phase nanoLC separation obtained for the different fractions.** (A) fraction 4 from [*PSI^+^*], (B) fraction 4 from [*psi^−^*] and (C) fraction 6 from [*PSI^+^*].
(TIF)

Figure S3 **Example of quantification result and validation.** (A) Quantification of spiked internal standards: BSA (ALBU_BOVIN) and Phosphorylase B (PHS2_RABIT). BSA was used for normalization between the samples. Phosphorylase B measures the technical replication between injections in the LC-MS system. (B) Example of multiplexed quantification data obtained, for two selected proteins, Actin and heat-shock proteins ssb1/ssb2, after BSA normalization of the data. (C) Western-blot validation of quantification using specific anti-actin or anti-Ssb1 antibodies. (D) Western-blot validation of identified chaperones using specific anti-Ssa, anti-Hsp104 and anti-Hsp82 antibodies.
(TIF)

Figure S4 **Qualitative comparison of proteins identified in the selected sucrose gradient fractions derived from an independent cell extract preparation and fractionation (biological replicates).** (A), (B) and (C) represent the distribution of the major biological process of each of the 121, 156 and 215 proteins identified in fractions 4B from [*psi^−^*] cells, fraction 4B from [*PSI^+^*] cells and fraction 6B from [*PSI^+^*] cells. The letter B refers to the replicates. (D) and (E) illustrate the qualitative comparison between fractions 4B [*psi^−^*] versus fraction 4B [*PSI^+^*] and fraction 4B [*PSI^+^*] versus fraction 6B [*PSI^+^*], respectively.
(TIF)

Figure S5 **Differential distribution of proteins for which concentrations differs between the selected fractions derived from an independent cell extract preparation and fractionation (biological replicates).** (A) and (D) correspond to the comparison of proteins identified in fraction 4B [*psi^−^*] versus fraction 4B [*PSI^+^*] and in fraction 4B [*PSI^+^*] versus fraction 6B [*PSI^+^*] respectively. (B) and (C) represent the distribution of the major biological processes attributed to proteins increased in fraction 4B [*psi^−^*] and fraction 4B [*PSI^+^*] respectively. 33 and 68 unique or significantly increased proteins are present within fractions 4B [*psi^−^*] and [*PSI^+^*], respectively. (E) and (F) represent the distribution of the major biological processes attributed to proteins increased in fraction 4B [*PSI^+^*] and fraction 6B [*PSI^+^*] respectively. 108 and 188 unique or significantly increased proteins are present within fractions 4B and 6B from [*PSI^+^*] cells, respectively.
(TIF)

Table S1 **Protein identification data for each replicate sample.** For each replicate sample and each identified protein, a table of identified peptides is given.
(XLSX)

Table S2 **List of proteins identified and quantified for each replicate sample.** Only non-redundant proteins identified in at least 2 of 3 replicates were quantified. The average amount of each protein is expressed in ng/µl. Ratios, Log ratios and two-way unpaired T-test of the fractions to be compared are given.
(XLSX)

Table S3 **List of proteins quantified in fractions 4 from [** ***psi^−^*** **] and [** ***PSI^+^*** **] cells.**
(XLSX)

Table S4 **List of proteins quantified in fractions 4 and 6 from [** ***PSI^+^*** **] cells.**
(XLSX)

Table S5 **List of proteins identified and quantified in an independent cell extract preparation and fractionation (biological replicates).** Table S5A: Protein identification data for each replicate sample. Table S5B: List of proteins identified and quantified for each replicate sample. Table S5C: List of proteins quantified in fractions 4B from [*psi^−^*] and [*PSI^+^*] cells. Table S5D: List of proteins quantified in fractions 4B and 6B from [*PSI^+^*] cells.
(XLSX)

Table S6 **Isoform filtering data obtained for the molecular chaperones Ssa1, Ssa2, Ssa3, Ssa4, Sse1 and Sse2.**
(XLSX)

File S1 **Data processing procedure for protein identification and quantification.**
(PDF)
