# Supplementary figures and images for: Qualitative and Quantitative Multiplexed Proteomic Analysis of Complex Yeast Protein Fractions That Modulate the Assembly of the Yeast Prion Sup35p
Source: PLoS One. 2011 Sep 13;6(9):e23659. doi: 10.1371/journal.pone.0023659 (PMC3172207; doi:10.1371/journal.pone.0023659)

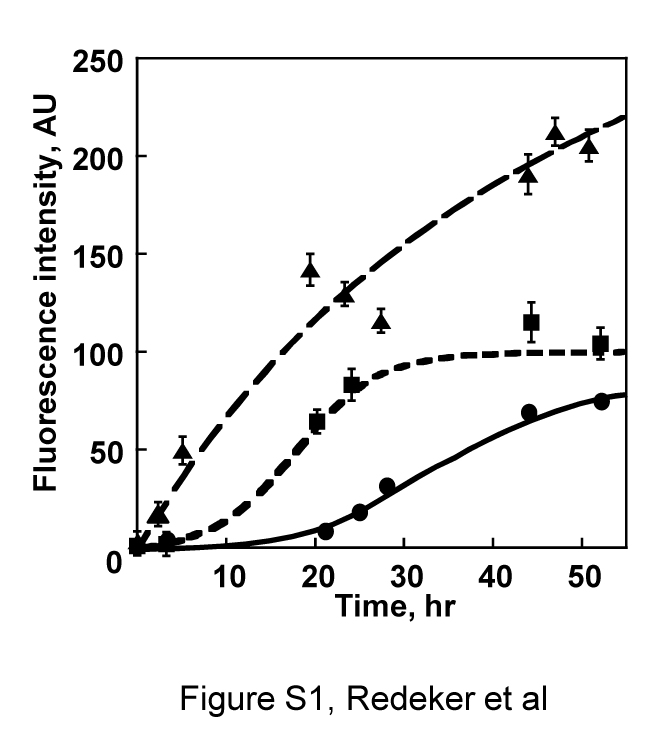

Supplement: Figure S1 — Assembly of full-length Sup35p into protein fibrils in the presence of cytosolic fractions from [PSI+] and [ psi− ] cells from an independent cell extract preparation and fractionation (biological replicates). Soluble Sup35p (7 µM) was incubated at 10°C in assembly buffer with no addition (solid circles) or containing 0.48 mg/ml of fraction 4B from [PSI+] (solid squares), or [psi−] (solid triangles) cells. The time course of Sup35p assembly in the presence of cytosolic fraction 6B from [PSI+] or [psi−] cells superimposes to that with no addition. (TIF) [file pone.0023659.s001.tif]

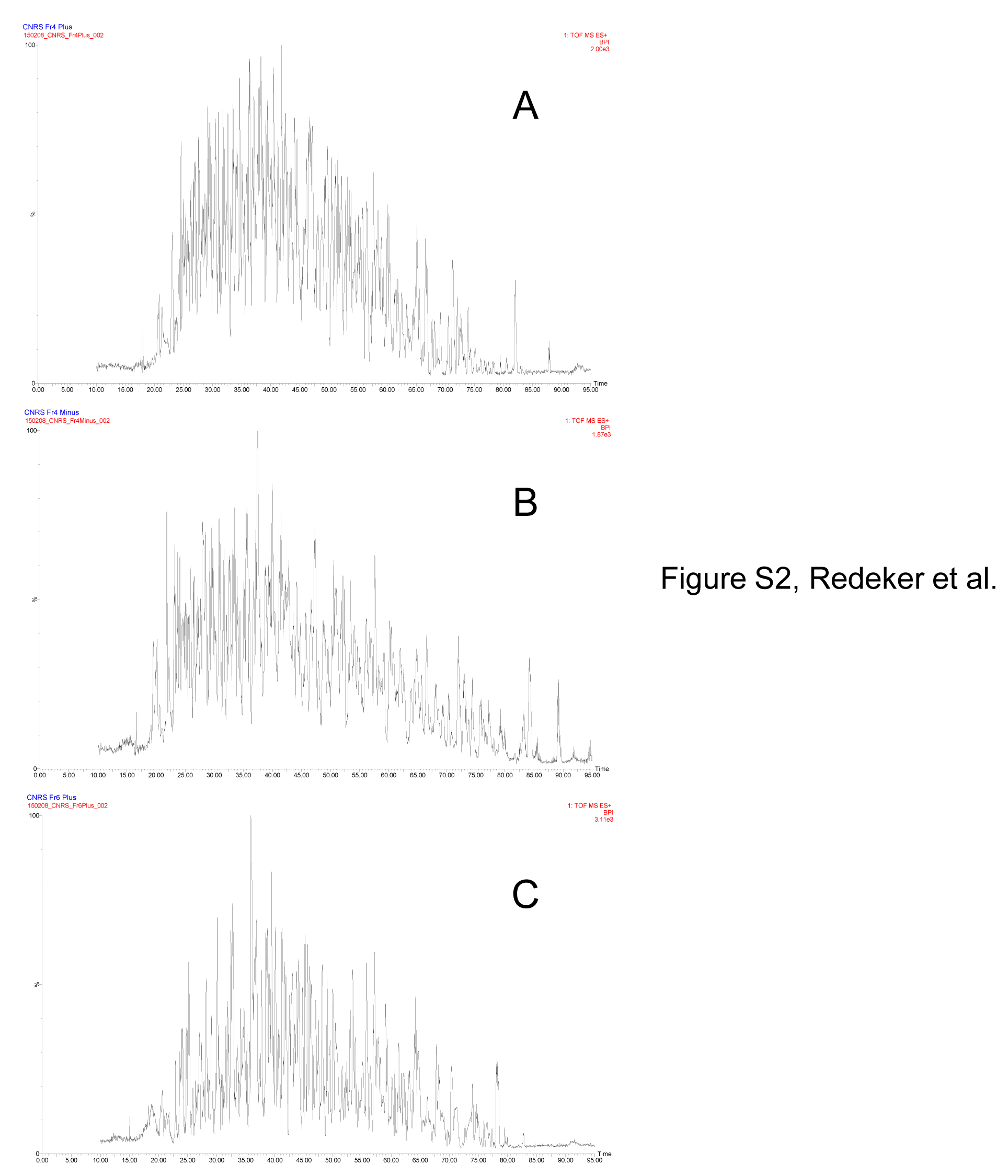

Supplement: Figure S2 — Reversed phase nanoLC separation obtained for the different fractions. (A) fraction 4 from [PSI+], (B) fraction 4 from [psi−] and (C) fraction 6 from [PSI+]. (TIF) [file pone.0023659.s002.tif]

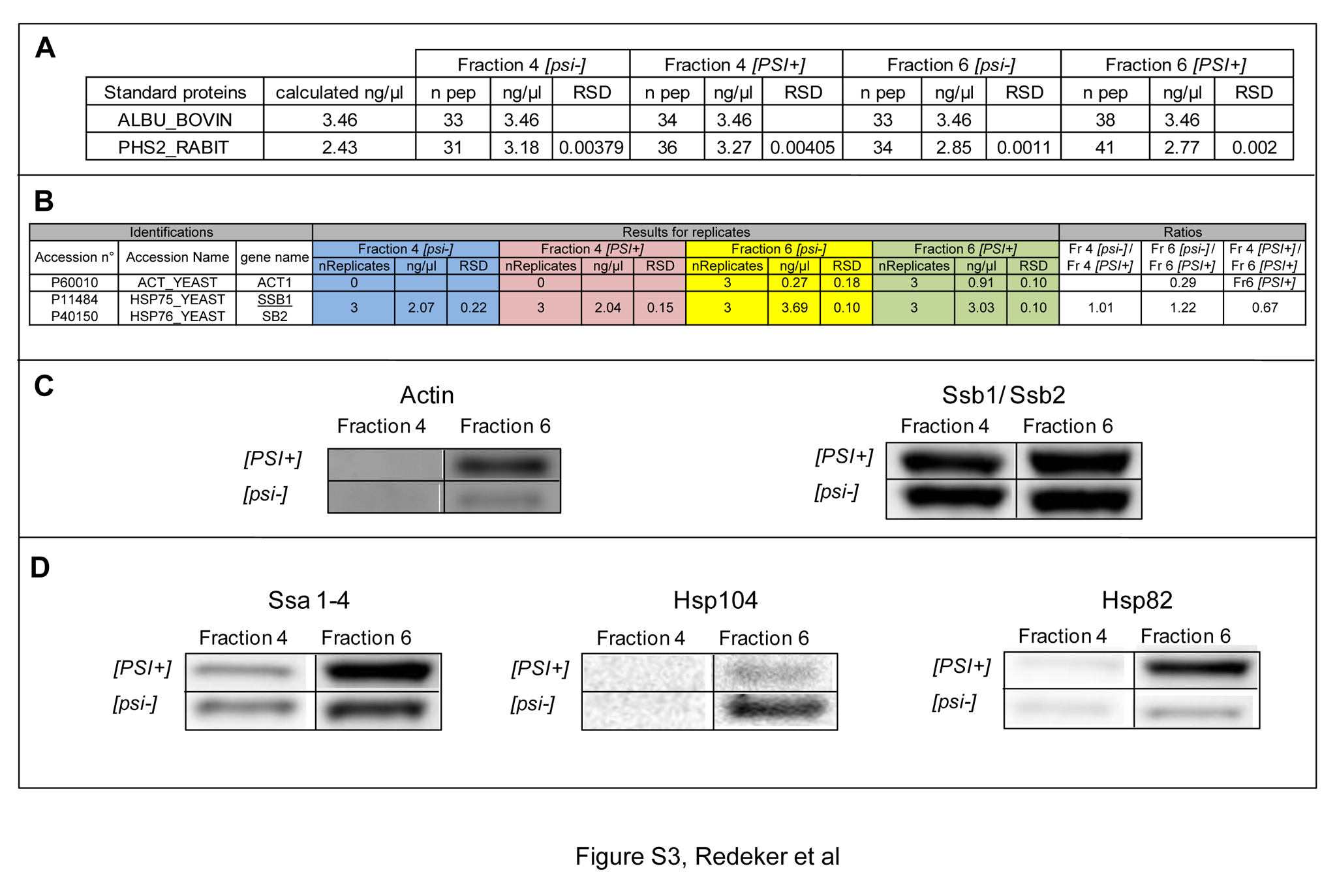

Supplement: Figure S3 — Example of quantification result and validation. (A) Quantification of spiked internal standards: BSA (ALBU_BOVIN) and Phosphorylase B (PHS2_RABIT). BSA was used for normalization between the samples. Phosphorylase B measures the technical replication between injections in the LC-MS system. (B) Example of multiplexed quantification data obtained, for two selected proteins, Actin and heat-shock proteins ssb1/ssb2, after BSA normalization of the data. (C) Western-blot validation of quantification using specific anti-actin or anti-Ssb1 antibodies. (D) Western-blot validation of identified chaperones using specific anti-Ssa, anti-Hsp104 and anti-Hsp82 antibodies. (TIF) [file pone.0023659.s003.tif]

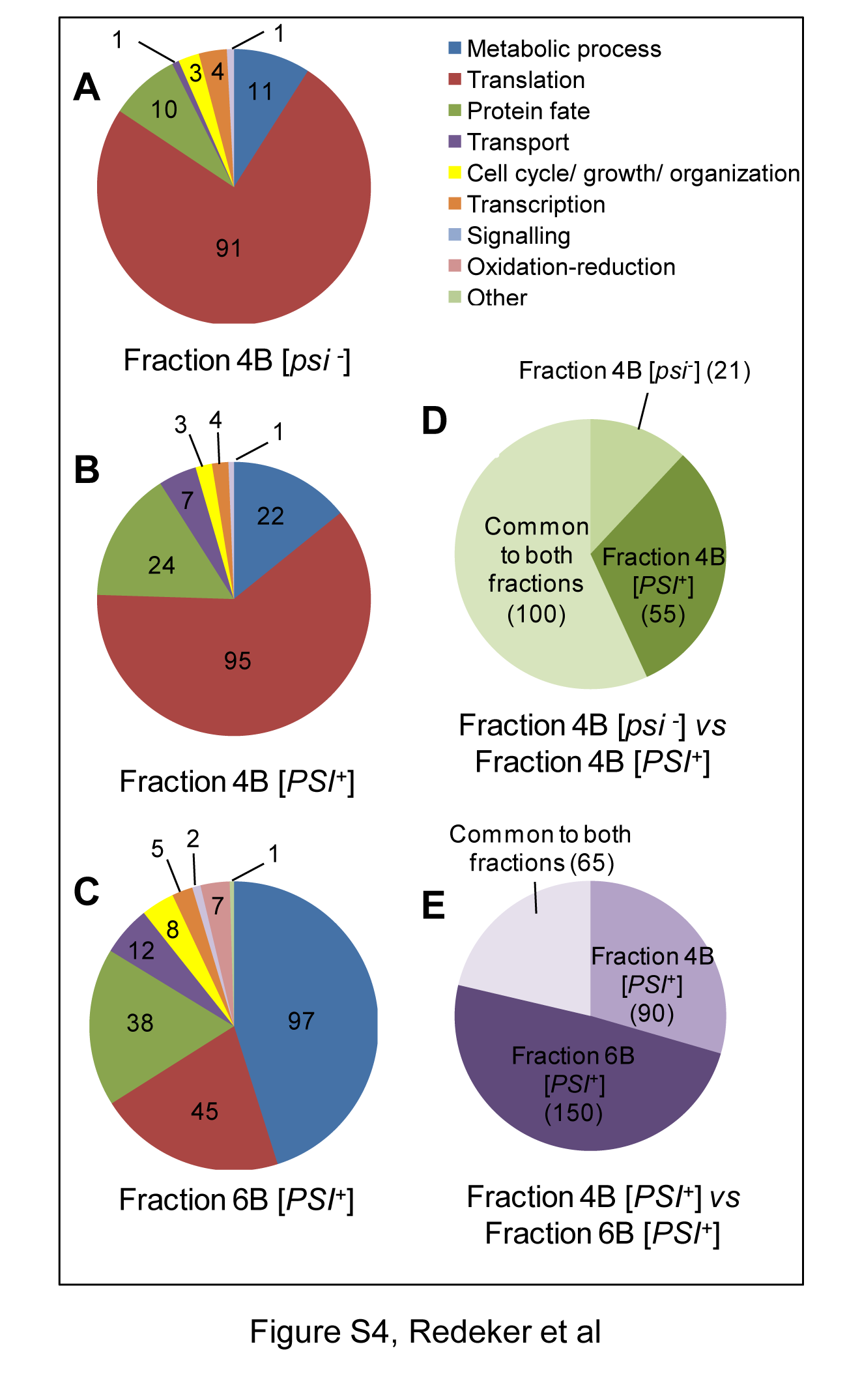

Supplement: Figure S4 — Qualitative comparison of proteins identified in the selected sucrose gradient fractions derived from an independent cell extract preparation and fractionation (biological replicates). (A), (B) and (C) represent the distribution of the major biological process of each of the 121, 156 and 215 proteins identified in fractions 4B from [psi−] cells, fraction 4B from [PSI+] cells and fraction 6B from [PSI+] cells. The letter B refers to the replicates. (D) and (E) illustrate the qualitative comparison between fractions 4B [psi−] versus fraction 4B [PSI+] and fraction 4B [PSI+] versus fraction 6B [PSI+], respectively. (TIF) [file pone.0023659.s004.tif]

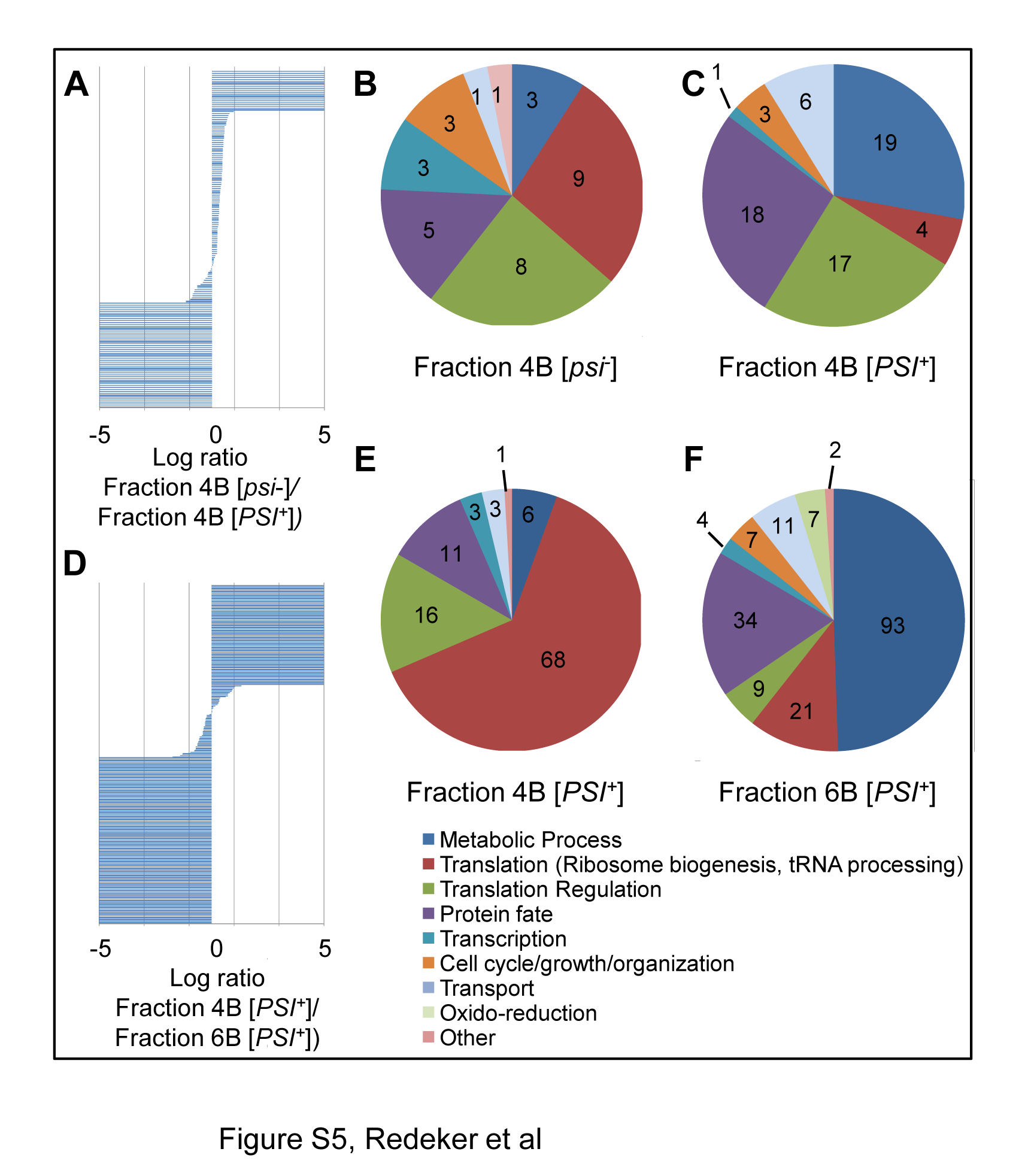

Supplement: Figure S5 — Differential distribution of proteins for which concentrations differs between the selected fractions derived from an independent cell extract preparation and fractionation (biological replicates). (A) and (D) correspond to the comparison of proteins identified in fraction 4B [psi−] versus fraction 4B [PSI+] and in fraction 4B [PSI+] versus fraction 6B [PSI+] respectively. (B) and (C) represent the distribution of the major biological processes attributed to proteins increased in fraction 4B [psi−] and fraction 4B [PSI+] respectively. 33 and 68 unique or significantly increased proteins are present within fractions 4B [psi−] and [PSI+], respectively. (E) and (F) represent the distribution of the major biological processes attributed to proteins increased in fraction 4B [PSI+] and fraction 6B [PSI+] respectively. 108 and 188 unique or significantly increased proteins are present within fractions 4B and 6B from [PSI+] cells, respectively. (TIF) [file pone.0023659.s005.tif]
