# Supplementary material for: Qualitative and Quantitative Multiplexed Proteomic Analysis of Complex Yeast Protein Fractions That Modulate the Assembly of the Yeast Prion Sup35p
Source: PLoS One. 2011 Sep 13;6(9):e23659. doi: 10.1371/journal.pone.0023659 (PMC3172207; doi:10.1371/journal.pone.0023659)
Supplement: File S1 — Data processing procedure for protein identification and quantification. (PDF) [file pone.0023659.s012.pdf]

File S1. Data processing procedure for protein identification and quantification using ProteinLynx Global SERVER (PLGS) v2.3 (Waters Corporation).

Identification of the proteins present in the selected fractions was performed with a strain (4932) specific yeast database (release 14.5 UniProtKB). After an assessment query, the software automatically sets the peptide and fragment mass tolerances. The final mass errors were found to be on average 3.8 ppm for the precursor and 12.1 ppm for the product ions, respectively, which is achieved by lock mass correction using the monoisotopic value 785.8426 of the double charged precursor mass of [Glu^1]-Fibrinopeptide B and operating the instrument at a resolution of at least 10,000 FWHM. Peptide and protein identifications were performed by setting the minimal number of fragment ion matches per peptide to 3 and that per protein to 7. The minimal peptide match per protein was initially set to 1, but final identification was only accepted with a minimum of 3 peptides per proteins (see also next section). No more than one missed trypsin cleavage was allowed. The tolerated modifications were cysteine carbamidomethylation, N-terminal acetylation, deamidation of asparagine and glutamine and oxidation of methionine.The protein false positive rate was set to 4%. The false positive rate was estimated using a randomized yeast database. Protein and peptide identification data are presented in Table S1. The results were further filtered. Only proteins identified with confidence i- by at least two constituting peptides and ii- in two out of the three LC-MS replicates were reported in Table S2. The use of replicate protein identification minimizes the false positive rate as false positive protein identifications, *i.e.*chemical noise, have a random nature and as such do not tend to replicate across injections. This approach rules out systematic search events errors due to the repeated ambiguity of spectra and the subsequent sequence assignment by a search algorithm as could be the case with peptide-centric searches. The final reporting protein false positive rate was found to be 0%. The list of the protein identified in this study is presented in Table S2.

Quantification of the identified proteins was achieved by a label-free approach, considering the intensity of the identified precursor ions. This approach involves integration of the total volume of each extracted, charged-state reduced, deisotoped and mass corrected ion across the mass spectrometric and chromatographic data using the embedded detection and deconvolution algorithm of the processing software [1, 2].The protein concentrations bound to the columns were estimated as described [3, 4]. Briefly, the average ion intensity of the three most abundant peptides related to a protein is standardized to that of an internal standard spiked into the sample at known concentration.This normalization can be conducted across the various fractions and offers the possibility to perform a meaningful multiplexed quantitative analysis across samples to be compared. Only proteins identified through at least three tryptic peptides and in at least

two LC-MS replicates were quantified for any given fraction. Proteins identified in at least two out of three replicates in one fraction but only in one out of three replicates in another fraction were considered as unambiguously identified for a single fraction, but not considered for relative quantification. Changes in protein concentrations were considered as significant when they differed by at least 40% (i.e. log values above 0.22 and below -0.22) in the fractions that are compared. This 40% difference was chosen as significance threshold because it was correlated to two-way unpaired T-test values below 0.05, representing a 95% probability that the distributions are different. For those exceptions where the T-test was higher, protein abundances were not considered as significantly different (Table S2).

The use of BSA as a digestion standard allows estimation of all experimental variations, including those due to digestion and injection [5]. The experimental variations were calculated by expressing the amount of BSA added before digestion and used for data normalization. Phosphorylase B tryptic peptides were used as injection standards. The recovery values are reported in Table S2 and were found to vary from 0.97 to 1.18. Also shown are the relative standard deviations, which were found to vary between 7 and 22%. These values are similar to those accounting for experimental variations (13 to 17%), without attempting to exclude statistical outliers, as median and average values, respectively, for the complete dataset. These error values allow assessment of the quantitative difference that could be identified within and between samples.

1. Levin Y, Wang L, Ingudomnukul E, Schwarz E, Baron-Cohen S et al. (2009) Real-time evaluation of experimental variation in large-scale LC-MS/MS-based quantitative proteomics of complex samples. J Chromatogr B Analyt Technol Biomed Life Sci 877: 1299-1305.
2. Li GZ, Vissers JP, Silva JC, Golick D, Gorenstein MV et al. (2009) Database searching and accounting of multiplexed precursor and product ion spectra from the data independent analysis of simple and complex peptide mixtures. Proteomics 9: 1696-1719.
3. Silva JC, Gorenstein MV, Li GZ, Vissers JP, Geromanos SJ (2006) Absolute quantification of proteins by LCMSE: a virtue of parallel MS acquisition. Mol Cell Proteomics 5: 144-156
4. Silva JC, Denny R, Dorschel C, Gorenstein MV, Li GZ et al. (2006) Simultaneous qualitative and quantitative analysis of the Escherichia coli proteome: a sweet tale. Mol Cell Proteomics 5: 589-607.
5. Levin Y, Jaros JA, Schwarz E, Bahn S (2010) Multidimensional protein fractionation of blood proteins coupled to data-independent nanoLC-MS/MS analysis. J Proteomics 73: 689-695.
